# Otolaryngology-Head and Neck Surgery clinical electives in undergraduate medicine: a cross-sectional observational study

**DOI:** 10.1186/s40463-022-00596-4

**Published:** 2022-11-12

**Authors:** Katrina M. Jaszkul, Marysia Grzybowski, Timothy Phillips

**Affiliations:** 1 Queen’s School of Medicine, Kingston, Canada; 2 University of Calgary’s Cummings School of Medicine, Calgary, Canada; 3 Department of Otolaryngology, Head and Neck Surgery, Kingston, Canada

**Keywords:** OHNS, Electives, Clerkship, Education, Oto-HNS, Student, COVID-19, Medical education

## Abstract

**Background:**

Otolaryngology-Head and Neck Surgery (OHNS) electives provide medical students opportunities for knowledge acquisition, mentorship, and career exploration. Given the importance of electives on medical student education, this study examines OHNS clinical electives prior to their cancellation in 2020 due to the COVID-19 pandemic.

**Methods:**

An anonymous 29-question electronic survey was created using the program “Qualtrics.” Themes included elective structure and organization, elective clinical and non-clinical teaching, evaluation of students, and the influence of electives on the Canadian Residency Match (CaRMS). The survey was distributed through the Canadian Society of Otolaryngology e-newsletter and e-mailed to all OHNS undergraduate and postgraduate program directors across Canada.

**Results:**

Forty-two responses were received. The vast majority of respondents felt that visiting electives were important and should return post-COVID-19 (97.6%). Most said they provide more in-depth or hands-on teaching (52.4% and 59.6%, respectively). However, there was great variability in the feedback, types of teaching and curriculum provided to elective students. It was estimated that 77% of current residents at the postgraduate program that responders were affiliated with participated in an elective at their program.

**Conclusions:**

Prior to the cancellation of visiting electives in 2020 due to the COVID-19 pandemic, electives played an important role in OHNS undergraduate medical education and career planning for students wishing to pursue a career in OHNS. Electives also provide the opportunity for the evaluation of students by OHNS postgraduate programs.

**Supplementary Information:**

The online version contains supplementary material available at 10.1186/s40463-022-00596-4.

## Background

Most Canadian medical schools do not have mandatory clerkship rotations in OHNS; therefore, for students interested in pursuing OHNS as a career, electives provide the opportunity to expand their clinical knowledge, explore OHNS, and demonstrate a keen interest in the field while visiting various programs [1]. Canadian OHNS electives have not been evaluated in the literature, and their structure and educational benefits remain unclear.

OHNS has traditionally been in the top five most competitive specialties in the Canadian Resident Matching Service (CaRMS) match [2]. Between 2013 and 2020, only two individuals were matched to the OHNS residency program without completing an elective and most individuals who matched completed more than three electives [3]. Performing electives at the candidate's desired program is likely extremely important: in 2019 and 2020, no applicant matched without an elective at their matched school [3].

Electives present several issues regarding their role in the Canadian undergraduate medical curriculum. For example, one barrier to electives is the cost to access the portal through the Association of Faculties of Medicine of Canada (AFMC), in addition to the cost of travel and housing to attend electives outside of their home institution [4]. Other challenges include the variability within electives, as each elective generally has its own objectives, feedback structure, and service to learning requirements.

Travelling electives (electives away from a student's home institution) were cancelled as of March 18, 2020, due to the COVID-19 pandemic and have not been approved for the graduating class of 2022. Since this decision, there has been an ongoing discussion about their value and how students and programs may evaluate electives in the future [5]. This cross-sectional observational study seeks to examine their status prior to their cancellation in 2020 due to the COVID-19 pandemic. The information gained could be used to improve OHNS electives in the future by establishing themes in the learning experiences for students on electives identifying areas of inconsistencies in OHNS curriculum across Canada.

## Methods

An online survey was created using Qualtrics© at Queen's University, Kingston, Ontario, Canada. This anonymous voluntary survey was sent to members of the Canadian Society of Otolaryngology-Head and Neck Surgery (CSOHNS) and the directors of OHNS undergraduate and postgraduate programs across Canada. The survey was sent to all active members of the CSOHNS on October 23, 2020, to 484 people and it was sent a second time on November 25, 2020 to 482 individuals. All respondents consented to their participation in this survey. The 29-question electronic survey on Qualtrics© consisted of four major themes: Elective Structure and Organization, Elective Clinical and Non-Clinical Teaching, Evaluation of Elective Students, and Electives and the Residency Match (Additional file 1). This survey asked yes/no, multiple-choice, and rank order questions. For a complete list of questions, please see the Additional file 1.

## Results

### Demographics

**Fig. 1:**
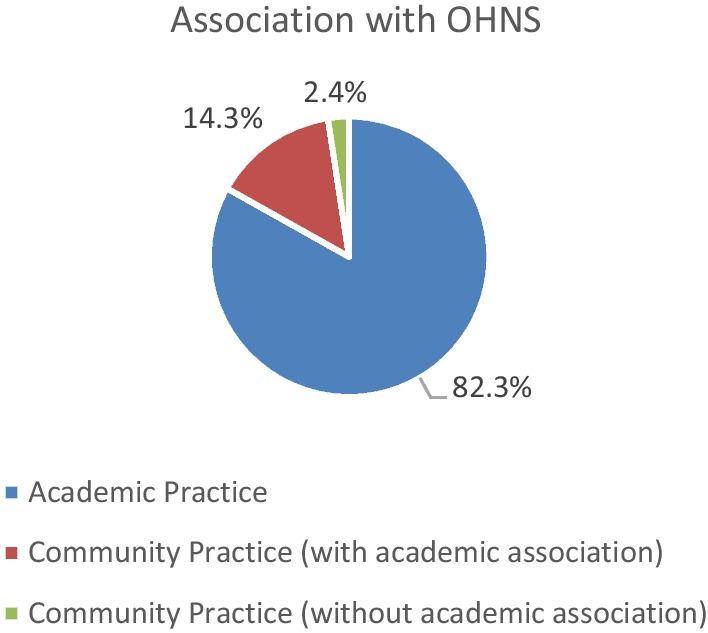
Figure depicting respondent affiliation/association to OHNS

A total of 42 surveys were completed. Of the respondents, 35 (83.3%) work in an academic practice, 6 (14.3%) practice in the community with an academic association, and 1 (2.4%) works in community practice with no academic association. Of the respondents, 38 (90.5%) have an OHNS residency program associated with their university and/or practice. 23.8% were UGME program directors, 9.5% were PGME program directors, and 50% contributed to the UGME/PGME without an official title. The majority of the responders did not have any extra training in medical education (85.4%), 7.3% have a Master of Education, and 4.9% had a Certificate or Diploma in Medical Education. Responses regarding a respondent's affiliated institution were not collected to help maintain the anonymity of this survey (Fig. 1).

### Elective structure and organization

On average, the respondents took 17.1 (SD 12.2) students per year to complete an elective at their centre with an average elective time of 2.3 (SD. 0.7) weeks. The respondents estimated that 69.1% (SD 26.9%) of these students planned to pursue a career in OHNS. The majority of responders (69.0%) stated that students work with multiple preceptors on electives, 28.6% responded that they are paired with a preceptor but work with others, and 2.4% reported that students work with one preceptor on their elective in a community center with an academic association.

Elective students typically spend most of their elective in the clinic and the operating room but may also perform duties related to inpatients. The expectation for elective students to join residents on call varied, with 14.3% of electives having mandatory call, 45.3% having non-mandatory call (but it is encouraged or expected), 28.6% of electives having non-mandatory call with no expectations, and 11.9% of electives do not have call.

### Elective clinical and non-clinical teaching

Typically, most electives do not have mandatory academic requirements (70.5%), while 20.5% require students to complete a formal presentation (e.g., Grand rounds, case report, teaching seminar, etc.), 6.8% provide written assessments (e.g., quiz, test), and 2.3% have some other form of academic requirement. On electives, some responders selected that they provide similar levels of teaching (47.6%) to elective students compared to medical students on mandatory rotations, and 52.4% provide higher levels of teaching. In addition, 59.5% of respondents will provide more hands-on experience to elective students compared to medical students on mandatory rotations, including suturing, nasopharyngoscopy, surgical incision, and FNA/biopsy (Table 1).

**Table 1:** Types of teaching sessions elective students get exposed to while on an away rotation

Type of teaching session	Percentage (%) of respondents that offer this learning experience
Sessions specifically designed for residents	30.1
Didactic teaching sessions specific for medical students	15.1
Specific assigned readings	12.9
Scheduled meetings with the program director	12.9
Simulations	8
Online computer-based sessions	7.5
Surgical videos	5.4

### Evaluation of elective students and electives

In terms of evaluation, 88% of respondents will ask medical students to evaluate their elective experience. The majority (61%) of the respondents offer pre-elective meetings to discuss objectives and expectations for the elective, and 74% of respondents offer post-elective meetings with the student to provide feedback. For students on electives, preceptors generally have higher expectations (73.8%), 19.1% of programs have similar expectations, and 7.1% of programs have lower expectations of their elective students (Tables 2, 3).

**Table 2:** Type of evaluation offered at the elective rotation site

Type of Evaluation Offered	Percentage of respondents that offer this format
Evaluation varies from evaluations provided by the student’s home institution	48.5
Evaluations specific to the elective	21.2
Informal evaluation face to face	22.7
Informal evaluation by e-mail	1.5

**Table 3:** The staff member who completes an elective student’s evaluation for an elective

Staff member	Percentage of respondents that offer this format
Multiple attending physicians	46.8
Residents	35.1
Administrative staff	7.8
Allied healthcare staff	6.5
A single attending physician	3.9

**Fig. 2:**
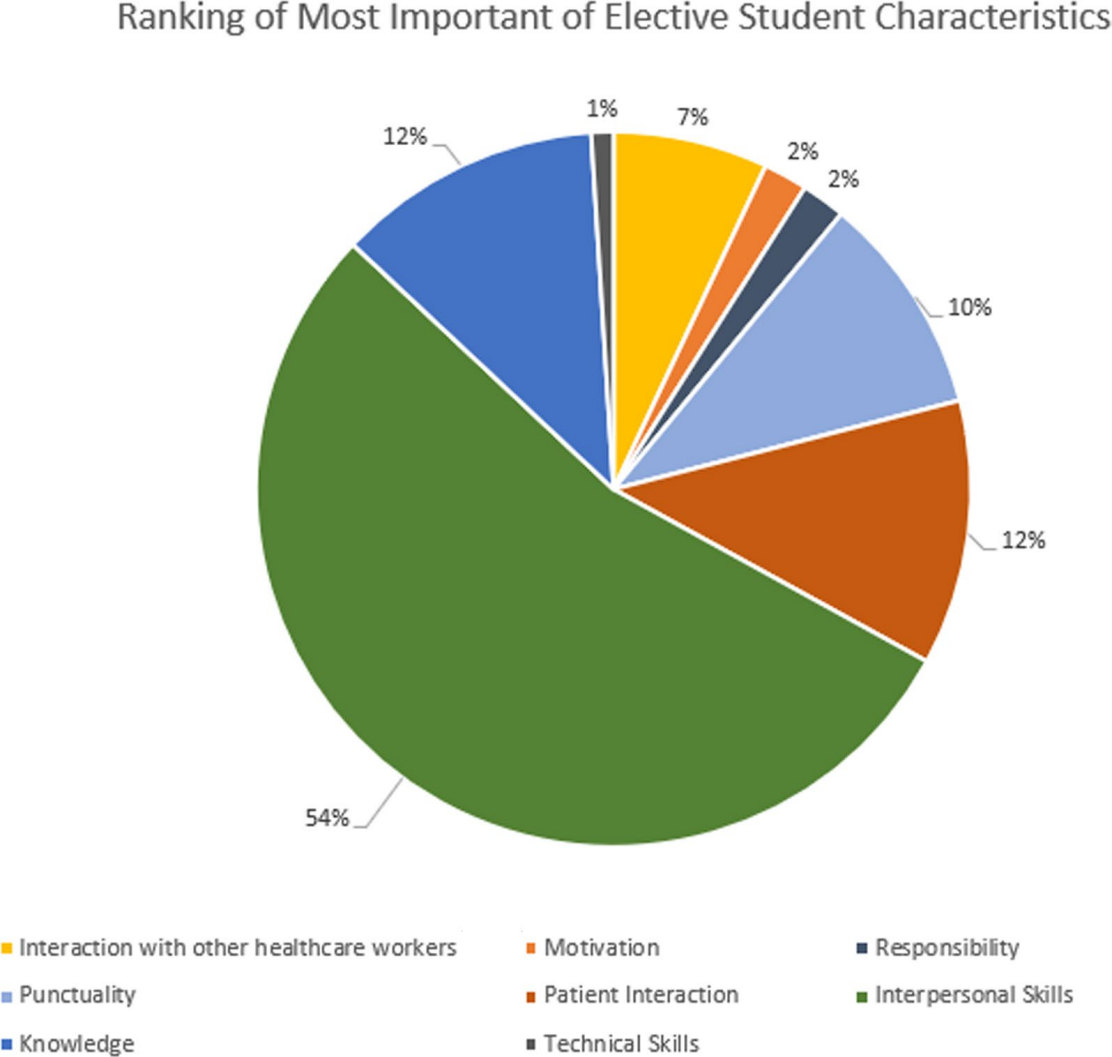
Summary of the order in which respondents ranked the most characteristic they look for in elective students

When asked to rank characteristics in elective students, these were overall the least to most important characteristics found in students: technical skills, punctuality, motivation, responsibility, knowledge, interactions with patients and the healthcare team members, and interpersonal skills (Fig. 2).

### Electives and the residency match

**Fig. 3:**
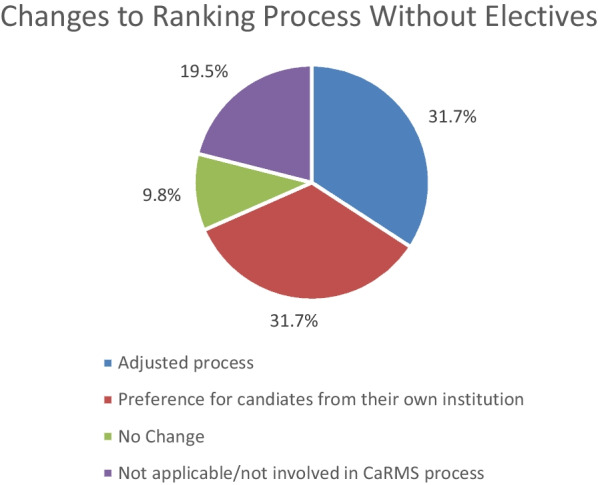
Figure depicting the changes being made to the CaRMS process as a result of the cancellation of away electives

We performed a sub-group analysis in which only the program directors were included, and 65% said it is one of the many factors that are looked at, and 35% said electives are important but not mandatory to obtain a residency interview; none responded that electives are mandatory to obtain a CaRMS interview. The mean proportion of current residents who completed an elective was 77.1%, and 97.6% reported hoping for electives to return after the COVID-19 pandemic. Due to the COVID-19 pandemic and lack of electives, 39.0% reported that their applicant rating process will be adjusted, 31.71% indicated they are more likely to select candidates from their own school, and 9.8% indicated their CaRMS selection process will not change (Fig. 3).

## Discussion

This anonymous online survey received responses from the majority of Canadian OHNS residency programs and represents an overview of clinical electives in OHNS in Canada prior to visiting electives being cancelled in 2020 due to the COVID-19 pandemic. Worldwide, the COVID-19 pandemic affected medical student education, particularly student electives, with some being cancelled altogether, some at a student’s home institution, and some electives became virtual [6]. However, the full long-term impact these changes have had on medical student education remains unclear [6]. In addition, these changes have affected students' transition from medical school to residency in terms of feelings of preparedness and confidence [7].

Even before the COVID-19 pandemic, this survey demonstrated that electives are quite variable from one institution to another, and there is a lack of consistency in the structure of electives across Canada, which is a common theme amongst surgical clerkship rotations across Canada [8]. This study provides insights into the value of visiting OHNS electives and its effects on the CaRMS process.

### Variability of structure and curriculum

Our survey demonstrated a variable amount of didactic learning, evaluation, procedural skills, and call expectations from one institution to another. This variation further contributes to the lack of consistency in the OHNS curriculum across Canada. This was depicted by the different amounts of time with various preceptors and areas of OHNS that elective students can partake in with variation in types of learning activities offered to the students. The majority of programs offered 2-week rotations for students and assigned students to spend most of their time in structured clinical activities (OR and clinic) compared with non-structured activities such as ward/inpatient responsibilities. With the similarities in their structure, it allows for the possibility of creating a more standardized structure for electives. When a standardized curriculum was implemented at a single institution in urology, it demonstrated that students were more likely to achieve core learning objectives [9].

Generally, when the format of fourth year electives was designed, their goal was to remain loosely structured to allow students to explore various careers pathways [10]. Providing structure to an elective enables students to put a greater focus on career selection, increasing clinical responsibilities expanding on clinical knowledge and skills, and decompressing from their core clerkship experiences [11]. Furthermore, there has been discussion about reforming electives in the USA to have a greater focus on student mentoring and career advising while on rotations [10]. With the current flexibility of the curriculum, it can allow students to focus on gaps in their knowledge and clinical skills, but this approach requires adequate guidance and support from the faculty at the elective site [10].

However, having a standardized rotation time would be beneficial for creating a tailored curriculum for elective students. This was demonstrated by the lack of consistency between elective sites can create barriers to student learning; this includes unknown expectations and the possibility of repetitive curriculum [12]. Additionally, this variation is an added barrier for students who are unable to do electives away from their home institution, as their elective experience will differ from their peers across the country. We feel it is also important to note that although performing inpatient and ward duties are important and can impart crucial knowledge, having time in the OR and clinic likely provides more educational opportunities and a chance for the students to be observed and receive feedback.

However, there is an advantage for a student having a variation in their elective experiences allows them to broaden their perspective of OHNS as a specialty. Additionally, being exposed to new learning environments can be beneficial for a student when deciding which institution best suits their learning needs when submitting their CaRMS rank order list. The differences in student experiences will likely not significantly affect the student by the end of their five-year surgical residency program.

### Elective clinical and non-clinical teaching

Electives are important for students to address knowledge gaps and learning goals as only 75% of Canadian medical schools teach OHNS-related lectures in pre-clerkship years, and the average Canadian medical student clerk will have 4.6 days of mandatory clinical clerkship experience within OHNS [13]. These knowledge gaps are not unique to OHNS and are common in other niche areas of medicine, such as ophthalmology, further highlighting the need for electives to help students develop their skills and knowledge in the field before postgraduate training [14]. However, beyond the clinical knowledge that will be further developed in residency, fourth-year electives offer the opportunity to teach students skills they will need in residency, including self-reflection, organizational skills, and professionalism from a variety of perspectives and experiences [15].

The majority of teaching sessions were designed for residents, so the value to medical students is debatable. Having educational content delivered specially for elective students would possibly help fill knowledge gaps. As responders appeared to be willing to provide more teaching to elective students, this may not be much of a challenge to convince the attending surgeons to implement these learning objectives in the elective curriculum. However, exposure to the types of learning offered to residents can be beneficial for students to assess the teaching styles offered at various institutions.

### Evaluation of elective students

In the Canadian curriculum, most instructional OHNS content outside of elective time did not contain opportunities for formal evaluation [16]. On the other hand, we found that the majority of our responders did provide an opportunity for evaluation, most commonly in a post-elective meeting (74%), and most responders evaluate students through a formal evaluation from (70%). These conversations can help set expectations for the student to focus their learning and for the program to evaluate how the student’s knowledge and abilities change over the elective. Pre-elective meetings are helpful for students to establish expectations with program directors, highlight learning goals, and ensure the program is aware of their baseline knowledge [17]. In addition, feedback can be helpful for students to discover areas of improvement and receive feedback on things they are doing well and helps the student become an active participant in their learning process [17, 18]. Interestingly, when a formal evaluation was used, it was most commonly the elective student's home evaluation form that was used. Having multiple different feedback forms used to evaluate students can lead to ambiguity when trying to compare students during CaRMS ranking. A standardized form used by all programs could be one method to decrease this variability.

Program directors tend to focus more on interpersonal skills and teamwork than academic excellence and technical skills. For example, in a study from the United States of America (USA), electives were seen as a valuable tool for programs to assess candidates, and they were seen as an audition to show the department a student’s interpersonal skills [19]. However, the program directors placed a higher value on knowledge and the interview process when asked to select the most important traits of a potential candidate [19]. Interpersonal skills are challenging to evaluate through any other format than an elective, as interviews may not represent a candidate's ability to collaborate or demonstrate an ability to work in team settings.

### Electives and the residency match

Prior to the pandemic, most Canadian residency programs placed a higher weight on performance during electives, letters of reference, and the interview process [20]. This survey depicted that most residents (77.4%) had done an elective at the institution in which they matched. Many programs found that electives are essential for determining if someone will match over interviews alone. Electives may be especially advantageous for a student without a home program as it helps them gain exposure, build connections, and confirm their career choice [21]. From the student perspective, electives are beneficial for students to determine which programs align best with their career goals, geographic preferences, and characteristics of the learning environment [20]. This may also demonstrate the importance of students without a home OHNS program having the opportunity to perform electives outside their institution to obtain a residency position. In a study from the USA, 78.8% of students without a program at their home institution felt like they were more disadvantaged in their chances of obtaining an OHNS residency compared to their colleagues from schools with a program [22]. With the current suspension of travelling electives, it poses challenges for students without OHNS programs at their home institutions to acquire meaningful letters of recommendation to comment on their abilities as a student [6].

These challenges have led both programs and students alike to find creative solutions to be able to mimic the traditional CaRMS experience as closely as possible given the virtual format [23]. One of the new ways to build relationships and allow students to meet the residents and the faculty has been the introduction of Zoom open houses, Zoom resident-only mixers, and more regular updates to program websites [23, 24]. As 31.7% of the survey respondents stated that there will be changes to the CaRMS process, it poses the question of how might these changes be made? In countries like the USA, even prior to the pandemic, the U.S Medical Licensing Exams (USMLE) scores and letters of recommendation were the two most important factors for a candidate to obtain a spot in a residency program [20]. While, in Canada, with the absence of objective measures of grades and scores, it is postulated that programs will begin to rely more heavily on other factors including, research, volunteerism, clerkship performance, and the virtual interview process, as a lack of travelling electives makes it difficult for programs to accurately assess a student’s clinical performance [20, 25].

### Limitations and commentary

There are several limitations to this study as it was a qualitative study. Not every Canadian OHNS program responded to this survey, and these results may not reflect the opinions of all programs. This may be especially true for provinces with only one to two programs, as candidate selection may vary from one province to another. There may have also been some response bias as some programs filled out this survey as they would have prior to the COVID-19 pandemic, while others completed the survey with the cancellation of electives in mind.

## Conclusion

This study demonstrates that electives provide valuable learning and career opportunities for senior medical students to gain further experience in OHNS and help prepare them for the CaRMS match. The majority of those who responded felt that electives should return post-COVID.

There is room for improving continuity in the evaluation and curriculum of electives across the OHNS programs in Canada. In the future, it would be ideal to create learning objectives and a curriculum that students could complete during their travelling electives. This would maximize the learning potential of visiting electives and facilitate continuous learning and skills development for students completing multiple OHNS electives. Some possible ideas for future studies to help address this would include creating standardized evaluation forms for OHNS electives, an online curriculum for students, or a set of competency-based objectives that students can try to meet during their electives (similar to the Royal College Competency by Design objectives).

As electives appear to play a clear role in students gaining OHNS residency spots, the authors would encourage the reinstatement of travelling medical electives to ensure equal opportunities for all medical students wishing to apply to OHNS and other specialties.

## Supplementary Information

**Additional file 1.** Table depicting the questions and responses from the survey.

## Data Availability

Data sharing is not applicable to this article as no datasets were generated or analysed during the current study.

## Abbreviations

AFMC: Association of Faculties of Medicine of Canada
CaRMS: Canadian resident matching services
COVID-19: Coronavirus disease/SARS-CoV-2 virus
CSOHNS: Canadian Society of Otolaryngology-Head and Neck Surgery
FNA: Fine needle aspiration
OHNS: Otolaryngology-Head and Neck Surgery
OR: Operating room
PGME: Postgraduate Medical Education
UGME: Undergraduate Medical Education
USA: United States of America
USMLE: U.S Medical Licensing Exams
