# Supplementary material for: Otolaryngology-Head and Neck Surgery clinical electives in undergraduate medicine: a cross-sectional observational study
Source: J Otolaryngol Head Neck Surg. 2022 Nov 12;51:42. doi: 10.1186/s40463-022-00596-4 (PMC9652593; doi:10.1186/s40463-022-00596-4)
Supplement: Supplementary file 1 — Additional file 1. Table depicting the questions and responses from the survey. [file 40463_2022_596_MOESM1_ESM.docx]

**Additional file 1**

Table depicting the questions and responses from the survey.

| **Question** | **# Responses** |
| --- | --- |
| **Demographics** |  |
| Is your practice in a community setting, or do you have an association with a university? | 42 |
| Is there an Oto-HNS residency program associated with your university or practice? | 42 |
| What is your role in your university UGME/PGME program? | 42 |
| Do you have any special training in medical education, i.e. Masters of Education or extra training? | 41 |
| How many students complete an elective in Oto-HNS per year at your center? | 42 |
| What percentage of these students do you believe are pursuing a career in Oto-HNS? | 42 |
| **Elective Structure and Organization** |  |
| How long is a typical OtoHNS elective at your institution? | 42 |
| Are medical students paired with an individual preceptor for the elective, or do they work with multiple preceptors? | 42 |
| How are elective students' schedules designed? | 47 |
| **Elective Clinical and Non-Clinical Teaching** |  |
| Please select if students are required to complete any of the following while on elective: a written assessment, a formal presentation, research project, no requirements, other. | 44 |
| Are there specific non-clinical education sessions for elective students (either optional or mandatory)? Please select all that apply | 93 |
| How do your expectations of elective medical students compare to those of medical students who are doing a mandatory rotation through Oto-HNS? | 42 |
| Do you feel that you provide more in-depth clinical teaching for elective students compared to other medical students? | 42 |
| Do you feel that you allow elective students to gain more hands-on experience compared to other medical students? | 42 |
| Please indicate how often you allow elective students to perform the technical skills: nasopharyngoscopy, microscopic ear debridement, surgical incision, suturing, FNA/biopsy | 35 |
| What are the on-call expectations for elective students (evenings, nights, or weekends)? | 42 |
| Where do students spend the majority of their time? Please ensure it adds up to 100% (operating room, clinic, performing duties related to inpatients, other) | 41 |
| **Evaluation of Elective Students** |  |
| Is there a pre-elective meeting with the student to discuss objectives and expectations? | 31 |
| If yes to the above question, who leads this meeting? |  |
| Is there a post-elective meeting with the student to provide feedback? | 42 |
| If yes to the above question, who leads this meeting? |  |
| How are elective students evaluated during their elective? Select all that apply. | 66 |
| Who contributes to the evaluation of the elective students? Select all that apply | 77 |
| Please rank the following characteristics 1-8 (1 being the most important) that you use to evaluate elective students. To adjust the rank list, drag and drop each row. (Interactions with other healthcare team members, motivation, punctuality, patient interaction, interpersonal skills, knowledge, technical skills) | 41 |
| **Electives and the Residency Match** |  |
| How important is it that students complete an elective in order for students to obtain a residency interview? | 41 |
| In your best estimate, what percentage of residents in your program completed an elective at your program as a medical student? Leave blank if your school does not have a residency program | 35 |
| Do you wish to see, if possible, Otolaryngology electives for medical students in the future? | 41 |
| Given the current cancellation of electives due to COVID, how do you feel this will affect your process in rating CARM's applicants? | 41 |
| Do you have any comments or suggestions for this survey? | 7 |
